# Dimensional variability characterization of additively manufactured lattice coupons

**DOI:** 10.1186/s41205-022-00141-z

**Published:** 2022-05-07

**Authors:** Kirstie Lane Snodderly, Magdalene Fogarasi, Yutika Badhe, Ankit Parikh, Daniel Porter, Albert Burchi, Laura Gilmour, Matthew Di Prima

**Affiliations:** 1 US Food and Drug Administration, White Oak, Silver Spring, Maryland USA; 2 Chenega Professional Services, Anchorage, Alaska USA; 3 Oak Ridge Institute for Science and Education, Oak Ridge, TN USA; 4 EOS North America, Novi, MI USA

**Keywords:** Accuracy, Lattice, Titanium, Powder bed fusion

## Abstract

**Background:**

Additive manufacturing (AM), commonly called 3D Printing (3DP), for medical devices is growing in popularity due to the technology’s ability to create complex geometries and patient-matched products. However, due to the process variabilities which can exist between 3DP systems, manufacturer workflows, and digital conversions, there may be variabilities among 3DP parts or between design files and final manufactured products. The overall goal of this project is to determine the dimensional variability of commercially obtained 3DP titanium lattice-containing test coupons and compare it to the original design files.

**Methods:**

This manuscript outlines the procedure used to measure dimensional variability of 3D Printed lattice coupons and analyze the differences in external dimensions and pore area when using laser and electron beam fabricated samples. The key dimensions measured were the bulk length, width, and depth using calipers. Strut thickness and pore area were assessed for the lattice components using optical imaging and µCT.

**Results:**

Results show a difference in dimensional measurement between printed parts and the computer-designed files for all groups analyzed including the internal lattice dimensions. Measurements of laser manufactured coupons varied from the nominal by less than 0.2 mm and results show averages greater than the nominal value for length, width, and depth dimensions. Measurements of Electron Beam Melting coupons varied between 0.4 mm-0.7 mm from the nominal value and showed average lengths below the nominal dimension while the width and depths were greater than the nominal values. The length dimensions of Laser Powder Bed Fusion samples appeared to be impacted by hot isostatic press more than the width and depth dimension. When lattice relative density was varied, there appeared to be little impact on the external dimensional variability for the as-printed state.

**Conclusions:**

Based on these results, we can conclude that there are relevant variations between designed files and printed parts. However, we cannot currently state if these results are clinically relevant and further testing needs to be conducted to apply these results to real-world situations.

## Introduction

Additive manufacturing (AM), commonly referred to as 3D Printing, has been widely adopted by the medical industry over the last decade due to its ability to make complex geometries such as lattice structures and parts individualized to a specific patient. This has led medical regulatory bodies, like the US Food and Drug Administration (FDA), to consider what the regulatory considerations are for the technology [1, 2] while still clearing a number of products for use [3]. While adoption of the technology continues, there is extensive work needed to create consensus standards to streamline the technology’s adoption rate and create more confidence in 3DP parts [4]. Understanding the dimensional variability of 3DP parts is a key design consideration to ensure 3DP products such as patient matched devices, anatomic models, cutting guides, and implants will have the appropriate dimensional fidelity for their intended use.

The design and 3D Printing process for medical devices, especially patient-matched workflows, may have many steps where errors can occur and propagate. In order to convert an original imaging file or computer aided drawing (CAD) to an acceptable file format compatible with the 3DP system, there may be multiple transfers and/or modifications of the design file through different software. These software workflows may modify the original design by rotating the part for proper orientation, fixing errors, and layer slicing to generate build paths. File data conversion has the potential to alter part dimensions and geometry as a result of software capabilities and algorithms [2]. In addition, digital build preparation choices such as part orientation, part placement within the build volume, and part packing density may affect the final product’s material properties, surface finish, and post-processing difficulty [1]. The FDA recommends that the 3DP system’s build volume should be validated to ensure consistent and acceptable properties within and between builds and that dimensional tolerance should be specified for the worst-case device [2].

The issue of 3DP variability has been studied through direct dimensional measurements of printed parts [5, 6], assessing the ability for 3DP parts to be assembled [7, 8], and through computational efforts to compensate for observed dimensional errors during their build process [9, 10]. Dimensional variability studies on coupons made using material extrusion approaches have shown an effect of build direction on variability [5, 6, 11] while others showed a dependence on build location [10] or feature size [12, 13]. Similar trends, if differing in magnitude, have also been observed for other 3DP processes like vat photopolymerization, powder bed fusion, and material jetting [5, 11, 12]. While all works showed dimensional variability and a deviation from the nominal (designed) dimensions, the variability was able to fit within existing grades of variability per ISO 286–1 and were within the range of other manufacturing methods [6, 12]. Given the desire for 3DP parts to be made and functional near-net shape, assessing the ability for parts to be assembled is akin to functional performance testing of the dimensional variability of 3DP parts. Rupal et al. proposed using two coupons designed to interlock via two posts and holes to assess the effect of 3DP variability on final part assembly [7]. Dantan et al. proposed a similar design using four posts and holes to computationally assess the effect of 3DP variability [8].

Direct measurement of 3DP dimensional variability is important, the ability to use computational tools to model systematic variability offers the potential to reduce final part deviation from nominal by accounting for it in the design stage. This can be done through simulating the slicing process and applying the expected material errors of flow and shrinkage [14] or performing detailed thermal modeling of the design and applying the material response via thermal expansion and linear elastic response [7]. Another approach is to model the “skin” of the 3DP part to predict how far from the nominal dimensional specification the part is expected to be [8, 9].

While computational modeling and coupon assessment methods can evaluate nominal variability in 3DP parts, direct device measurements may better evaluate the unique design parameters for 3DP medical applications. Toth et al. used micro-computed tomography (μCT) and a coordinate measuring machine (CMM) to assess the dimensional deviation from nominal in an acetabular implant [15] while Jin et al. compared the dimensional variability between different 3DP technologies using stone dental molds [16]. For cranial models, Salmi et al. has compared the accuracy of different 3DP technologies using CMM and μCT [17]. While most of these efforts have been focused on external dimensional accuracy and variability, medical devices often have designed porosity/lattice structures that have been assessed via μCT for dimensional accuracy [18, 19].

These previous works have addressed dimensional variability on either coupons, molds, models, or polymer structures. However, they have not addressed variability on metallic medical implant-like structures. These structures often have solid and porous components and complex designs which were not comprehensively investigated in the previous studies. Assessing the variability of a more representative medical device coupon will provide a better understanding to device designers and regulators on the expected variability in medical devices.

This study seeks to use test coupons representative of a medical device commonly made using 3DP, the intervertebral body fusion device, often known as a spinal fusion cage. The first part of this study investigated the effect of design and 3DP technology type by assessing framed and frameless lattice spine cage coupons made using laser and electron beam powder bed fusion and the effect of hot isostatic press post processing (Aim 1). The second part of the study was to assess the effect of the lattice relative density on the dimensional variability of bulk and lattice dimensions and the effect of hot isostatic press post processing (Aim 2). For this part, frameless cages made using laser powder bed fusion with four different designed lattice densities were measured. To further investigate the challenges of measuring 3DP lattices, additional cylindrical lattice coupons made using laser powder bed fusion were assessed using μCT and optical measurement techniques (Aim 3).

## Materials and methods

### Sample generation

AM lattice coupons were designed using general spine cage features. The basic shape was designed in SolidWorks 2019 (Dassault Systèmes; Waltham, Massachusetts). To create the lattice structure, large cubes of lattice were generated with a Body Centered Cubic (BCC) unit cell type and relative densities ranging from 0.15 to 0.45 in Materialise Magics 25.0 (Materialise; Leuven, Belgium). The basic spine cage shape was first imported into Magics and positioned inside the lattice cube and then a Boolean intersection operation was performed to convert the relevant part of the coupon to the lattice structure. In addition to varying the relative density of the coupons, the outer contour was either left solid (frame) or as a lattice (frameless).

### Experimental groups

**Fig. 1:**
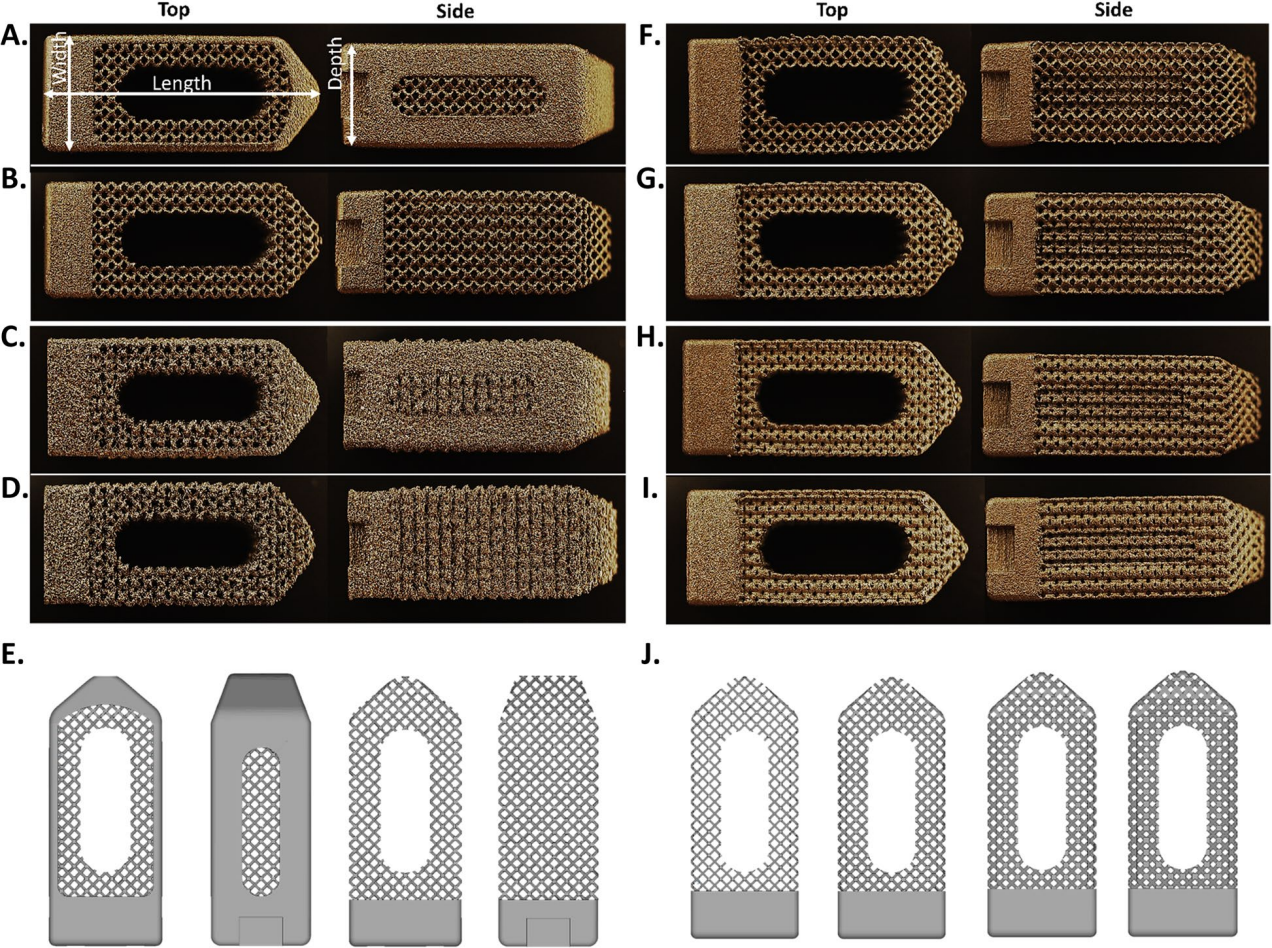
The test groups are: (**A**) Laser 1 with Frame, (**B**) Laser 1 without Frame, (**C**) EBM with Frame, (**D**) EBM without Frame, (**E**) CAD Design file for groups with and without frames, (**F**) Laser 2 with 15% Relative Density, (**G**) Laser 2 with 25% Relative Density, (**H**) Laser 2 with 35% Relative Density, (**I**) Laser 2 with 45% Relative Density, (**J**) CAD Design file for Relative Density groups (15%-45%)

Aim 1 of this study was to evaluate the dimensional variability across coupons with and without external frames across two different commercially sourced printing methodologies: Laser-based powder bed fusion (PBF-LB) using a ProX DMP 320 (3D Systems, Colorado, USA) referred to as Laser 1 and electron beam melting (EBM) using an Arcam A1 (Arcam, Sweden) (Fig. 1 A-D). For this aim, four sets of samples, two for each printing method, were manufactured using Titanium-6Al-4 V (grade 5). Laser 1 samples had external nominal dimensions of 22 × 9 × 8 mm with a frame (*n* = 20) and without a frame (*n* = 20). Additionally, electron beam melting samples had identical nominal dimensions with a frame (*n* = 20) and without a frame (*n* = 20). The framed and unframed design files were consistent across printing methods (Fig. 1E). One common post-processing step for 3D Printed medical devices is the use of Hot Isostatic Press (HIP) to relieve internal stress, improve the microstructure, and consolidate internal voids [20]. Therefore, after bulk measurements were performed, half of each of the previously described groups were randomly selected for HIP processing per §13.1.1 of ASTM F2924 – 14 “Standard Specification for Additive Manufacturing Titanium-6 Aluminum-4 Vanadium with Powder Bed Fusion.”

Aim 2 of this study looked to evaluate the effect of lattice relative density (RD) on dimensional variability. A second laser company (Laser 2) was used to fabricate unframed lattice coupons using a Concept Laser M2 (Concept Laser, Germany) which then had their dimensional variability assessed across changes in lattice relative density (Fig. 1 F-J). The external dimensions were 22 × 9 × 8 mm and the relative density groups were: RD 15% (*n* = 10), RD 25% (*n* = 10), RD 35% (*n* = 10), and RD 45% (*n* = 10). The same HIP process from Aim 1 was used for this aim.

The goal of aim 3 is to further investigate the challenges of measuring 3DP lattices. Cylindrical lattice samples 20 mm in height and 15 mm in diameter made from Titanium-6Al-4 V (grade 5) were manufactured on an EOS M290 (EOS North America; Texas, USA) (Laser 3). The target relative density was 25% with a targeted strut thickness of 300 µm. The two lattice designs were truncated octahedron and stochastic Voronoi Tessellation Method (VTM).

### Bulk measurements: caliper measurements

For each lattice coupon for all three manufacturing types before and after HIP, 6″ digital calipers (Pittsburgh; California, USA) were used by a single operator to obtain five measurements for each dimension (length, width, and depth) at various locations along the sides (Fig. 1A) with the first measurement of the width and depth obtained from the solid base of the lattice coupon for each sample and the remaining four measurements obtained throughout the length of the lattice sample. All measurements were recorded, and averages and standard deviations were calculated for each sample, each test group, and each printing methodology.

### Lattice measurements: optical imaging

All groups were imaged using a HiRox RH-2000 microscope, a MXB-2500REZ Zoom Lens at 35X (4.26 μm resolution), and RH-2000 Ver 2.0.40 software (Hirox Co Ltd.; Tokyo, Japan). Side and top views were imaged for each group and are defined in Fig. 1.

### Lattice measurements: pore area analysis

For aim 1 test groups, an ImageJ (NIH; USA) particle analysis method was used to measure the pore area. For each image, a constant size rectangle was created to define the analysis area and crop out any incomplete pores. This rectangle had the same dimensions for all samples. Thresholding from 8-bit greyscale images was used to create a binary image. A segmentation threshold of 50% was applied for each side view image and a segmentation threshold of 70% was applied for each top view image. To obtain major and minor axes values for pore ellipsis, an ImageJ particle analysis plugin was used with a size range of 50,000—200,000 μm^2^ since the ideal pore size is 186,349 μm^2^.

For aim 2 test groups, an ImageJ particle analysis method was used to measure the pore area. Again, the same size rectangle was used for all samples and images were converted to 8-bit greyscale. A segmentation threshold of 60% or 80% was applied for each side view image and a segmentation threshold of 80% was applied for each top view image. The size range parameters for the particle analysis were 20,000—250,000μm^2^ for all relative densities. The ideal pore size differed for each relative density: RD 15% = 220,893 μm^2^, RD 25% = 165,915 μm^2^, RD 35% = 136,699 μm^2^, and RD 45% = 102,141 μm^2^.

For each image, an ellipse was fit to each pore and the major and minor axes were recorded. An outlier test based on the axes data distribution was performed to eliminate any data points outside of 1.5 times the interquartile range. The standard deviation and the resulting average ellipse area were calculated for each group. The error (standard deviation) for each axis measurement was propagated through the ellipse calculations and this propagated error is shown as error bars on the pore area graphs.

### Lattice measurements: strut thickness analysis—micro-computer tomography (μCT)

Micro-computer tomography (MicroCT) was used to measure the mean strut thickness of all the samples. All samples were scanned with a ScanCo Medical μCT100 (ScanCo Medical; Brüttisellen, Switzerland). Scanning parameters chosen were a voltage of 90 kVp, a beam current of 200 μA, an integration time of 350 ms, and a 0.1 mm copper filter. Isotropic image voxels were 10 μm in size. A 6.45 mm section of each specimen was imaged.

Post scanning, contours which define the analysis volume were manually defined on the axial slices for each sample, avoiding the test coupon frames for samples with frames. A fixed threshold value was chosen to differentiate the struts and the porosity in the lattice. A pre-set morphometry evaluation program in the ScanCo MicroCT software that implements a Direct Transformation (DT) mapping is used to quantify the average strut thickness values of each sample. The evaluation uses a model-independent assessment of the three-dimensional images to estimate 3-D morphometry parameters like structure thickness.

### Lattice measurements: strut thickness analysis—stereological evaluation

ASTM F1854-15: Standard Test Method for Stereological Evaluation of Porous Coatings on Medical Implants was used to measure strut thicknesses of cylindrical lattice coupons (Laser 3). Lattice samples were mounted in epoxy compound by embedding them perpendicular to the plane of the substrate and cured for 24 h. The resin was degassed prior to curing. A section was cut perpendicular to the surface and the sectioned face was polished and cleaned. High resolution optical images were obtained using a HiRox RH-2000 microscope at 50X magnification in dark field mode. The images were processed using ImageJ.

The images were converted to greyscale and then binary format using an automatic thresholding routine similar to the lattice pore area measurements. The image processing software was used to generate automatic void volume percentage (${V}_{v}$). According to ASTM F1854, horizontal grid lines were overlaid on the images and intersections along the grid lines were counted manually. Each time the grid line goes from either solid to void or void to solid was counted as one intersection. The number of intersections is twice the number of intercepts (${N}_{v}$). An estimate of the strut diameter ${(L}_{v}$) was calculated using the following expression that takes into consideration total length of lines (${L}_{T}$),${N}_{v}$, the magnification (M), and the previously calculated ${V}_{v}$.

$${L}_{v}= \frac{\frac{{V}_{v}}{100} \times \frac{{L}_{T}}{M}}{{N}_{v}}$$

Three images of each cross section were taken at different regions and processed; the average of these three measurements is reported.

### Statistical analysis

Analysis between coupon groups was performed using RStudio (RStudioTeam, Boston, MA; R Version 4.0.3). Variance between groups was assessed using ANOVA (α = 0.05) and Tukey’s HSD post hoc analysis (α = 0.05).

## Results

**Fig. 2:**
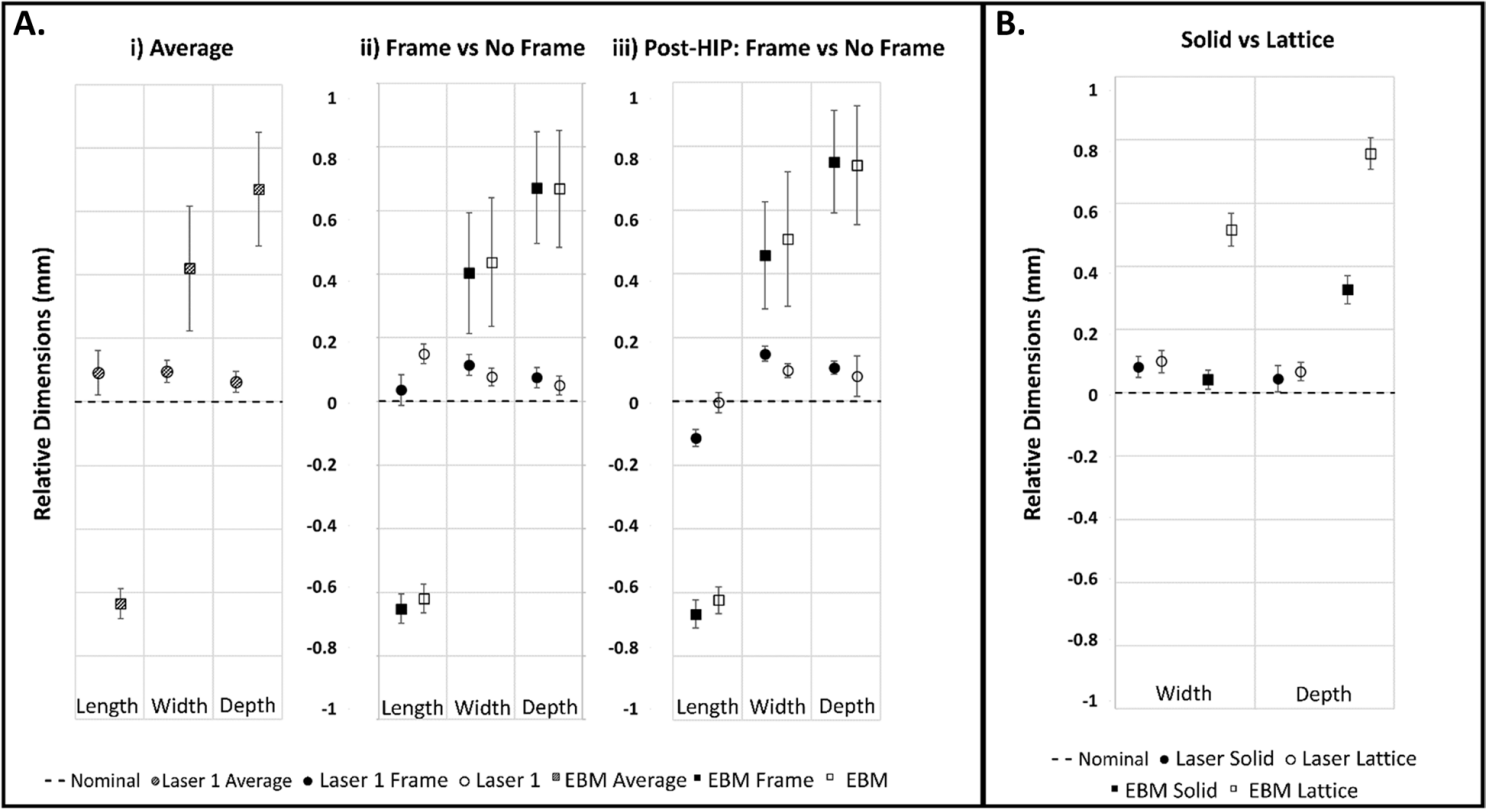
External coupon dimensions were quantified relative to the nominal by subtracting the nominal design values for length (22 mm), width (9 mm) and depth (8 mm) from the caliper measurements. **A** The average nominal values of all groups combined (*n* = 40) (i), the comparison between the frame and no frame groups (*n* = 20) (ii), and the post-HIP comparison between the frame and no frame groups (*n* = 10) (iii) are shown. Legend labels with “frame” refer to samples containing a frame. **B** External dimensions were also compared between the solid portion and the lattice portion of each sample

Figure 2 shows length, width, and depth averages which were calculated for each analysis group. The difference from nominal (length = 22 mm, width = 9 mm, depth = 8 mm) was quantified and compared across groups. Results were averaged for all test groups within each printer group (*n* = 40). All Laser 1 measurements varied from the nominal by less than 0.2 mm and results show averages greater than the nominal value for length, width, and depth dimensions (Fig. 2A(i)). The EBM measurements varied between 0.4 mm-0.7 mm from the nominal value and showed average lengths below the nominal dimension while the width and depths were greater than the nominal values.

The Laser 1 and EBM printing methods each contain two groups, one with an external frame and one without an external frame. Length, width, and depth averages were calculated for each test group and quantified relative to the respective nominal dimension. As shown in Fig. 2A.ii, most experimental groups differed from the nominal; the minimum average difference was the framed Laser 1 length measurement at 35 μm over the total 22 mm length while the maximum average difference occurred in the unframed EBM depth at 669 μm over the total 8 mm depth. In addition, there is a statistical difference (*p* < 0.05, Tukey’s HSD) between framed and unframed groups in all three external dimensions for the Laser 1 samples. For the EBM samples, there was only a statistical difference (*p* < 0.05, Tukey’s HSD) between the framed and unframed coupons for the width dimension. Both the length (*p* = 0.066, ANOVA) and the depth (*p* = 0.454, ANOVA) measurements did not show statistical differences between framed and unframed groups. The EBM width was approximately 0.41 mm greater than nominal and the depth was approximately 0.67 mm greater than the nominal for both groups. Similar to the Laser 1 groups, the EBM groups all differed from the nominal and did not show any preferences between framed and unframed groups.

To evaluate the influence of the HIP process on dimensional variability of 3D Printed lattice coupons, half of all samples were post-processed using a HIP method (Fig. 2A(iii)). All length, width, and depth measurements were obtained and plotted as stated previously. Trends in the data appeared consistent with the as-printed samples with the largest variations occurring in the Laser 1 length samples where the difference between the as-printed and post-HIP means was 150 μm. The width and depth change were less than 30 μm. The HIP process had a statistically significant (p < 0.05, Tukey’s HSD) effect on most of the external dimension measurements for Laser 1 samples. In samples with a frame, only the depth measurement did not show a statistically significant difference between the as-printed and HIP states (p = 0.241, Tukey’s HSD). For Laser 1 samples without a frame, all external dimensions showed a statistically significant difference post-HIP (*p* < 0.05, Tukey’s HSD). The EBM samples had a difference of approximately 10 μm in length, 63 μm in width, and 76 μm in depth between as-printed and HIP states. Statistical analysis showed that the EBM samples had a statistically significant difference after the HIP process for the width and depth measurements for both the framed and unframed groups (*p* < 0.05, Tukey’s HSD).

To isolate the solid portion from the influence of the lattice structure dimensional deviations, the first measurement of the width and depth was analyzed separately from the remaining measurements for each sample. It was found that there is a difference between the average first measurements and the remaining measurements for the width and depth dimensions for both Laser 1 and EBM groups (Fig. 2B). The Laser 1 measurements for width and depth showed a very small difference between the averages with all averages for solid and lattice below 0.1 mm. However, the EBM groups had the largest average dimensional difference between the solid and lattice components. For the width dimension, the solid was less than 0.05 mm above the nominal value while the lattice portion had greater than 0.5 mm average difference above the nominal. In the depth dimension, the solid portion was farther from the nominal than the other solid groups, but the lattice portion was approximately 0.75 mm greater than the nominal.

**Fig. 3:**
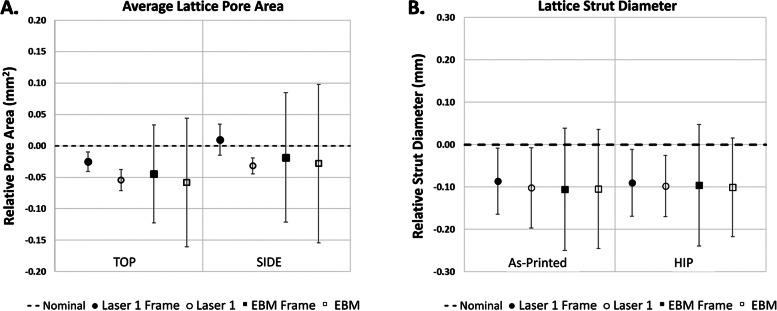
The pore area (**A**) was measured using optical imaging and ImageJ, and the strut diameter (**B**) was measured using micro-computed tomography

Images were taken of the top and the side of each sample (Fig. 1) using a HiRox Microscope and the lattice regions were evaluated for pore area using ImageJ. Data was quantified relative to the respective designed pore area for each group by subtracting the nominal designed pore area from the experimental value (Fig. 3A). Trends in the data show the pores tend to be smaller than the designed geometry, and EBM test groups show large variability in pore size for both the top face and the side face of the lattice coupons.

Three samples of each group were evaluated using a microCT system. Strut thickness values were quantified relative to the designed strut thickness for the corresponding group (Fig. 3B). Results indicate the average strut diameter was smaller than the nominal design, however, the variability was relatively consistent between all groups.

**Fig. 4:**
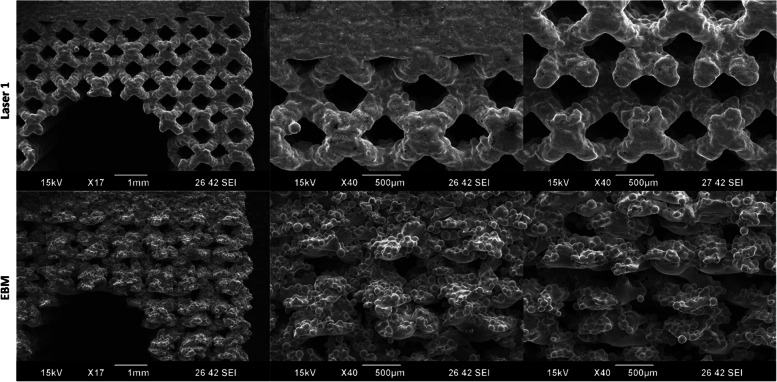
SEM Images of the same coupon design printed with the Laser 1 and EBM systems

As shown in Fig. 4, there are visible structural differences in the lattice coupon between the two printing methods when the same design file was printed. It is important to note that there were challenges in analyzing pore area in the EBM samples when using the non-destructive visual assessments shown in Fig. 3A.

**Fig. 5:**
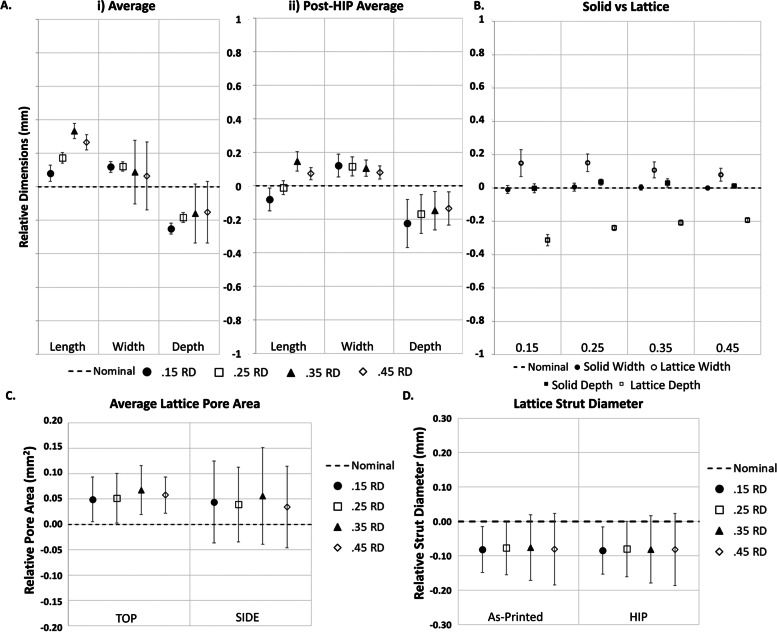
The effect of relative density on coupon dimensions was also examined. External dimensions were measured and subtracted from the nominal values for length (22 mm), width (9 mm) and depth (8 mm). **A** The average nominal values of all as-printed groups (*n* = 10) (i) and the average nominal values of the post-HIP groups (*n* = 5) (ii) are shown. **B** External dimensions were also compared between the solid portion of the sample and the lattice portion. **C** The pore area was measured using optical imaging and ImageJ, and (D) the strut diameter was measured using micro-computed tomography

Additional samples were created to evaluate the effect of relative density on dimensional variability. Samples were designed with varying relative densities (15%, 25%, 35%, 45%) and manufactured on a separate laser system (Laser 2) than the Laser 1 samples discussed previously. However, similar measurement techniques were applied to these samples. External length, width, and depth measurements were obtained for each relative density and quantified relative to the respective nominal dimension: length = 22 mm, width = 9 mm, depth = 8 mm (Fig. 5A(i)) by subtracting the nominal. There was a statistically significant difference in the length dimensions when the different relative densities were compared (*p* < 0.05, ANOVA). However, post-hoc analysis of as-printed samples only showed a statistical difference between the 15% and 35% length dimensions (*p* = 0.03, Tukey’s HSD).

To evaluate the influence of the HIP process on dimensional variability, length, width, and depth measurements were again obtained (Fig. 5A(ii)). There was a statistically significant difference for all three dimensions as a function of relative density. For the length dimension, the 15% and 25% RD samples did not show a statistically significant difference in the Tukey’s HSD analysis (*p* = 0.053, Tukey’s HSD), while all other length comparisons were statistically significant (*p* < 0.05, Tukey’s HSD). When comparing the width dimension, there was not a statistically significant difference between the 15% and 25%, 15% and 35%, and the 25% and 35% groups (*p* > 0.05, Tukey’s HSD). For the depth measurements, there was not a statistically significant difference between the 25% and 35% and the 35% and 45% groups (*p* > 0.05, Tukey’s HSD).

Trends for these samples mimicked those of the as-printed samples. When comparing the HIP dimensions to the as-printed state, there were no statistically significant differences found (*p* > 0.05, ANOVA). However, the length measurements appeared to cluster more closely to the nominal in the HIP state (< 0.2 mm) than the as-printed state (< 0.4 mm).

Similar to the printing method groups shown previously, data was compiled into two additional groups: the first measurement from each sample and the remaining measurements for each sample to represent the solid portion and the lattice portion of the sample, respectively. Width and depth measurements were obtained, and it appeared that there is a visible difference between the solid and lattice measurements for width and depth measurements for all relative densities (Fig. 5B) with the solids closer to nominal, the width larger than nominal, and the depth smaller than nominal.

The top and the side of each sample were imaged, and ImageJ was used to evaluate pore size. Data was quantified compared to the designed pore area for each group (Fig. 5C). Trends in the data show that the pores tend to be larger than the designed geometry, but the variability in pore size for both the top face and the side face of the lattice coupons did not appear to vary between relative density groups.

Three samples of each group were evaluated using a microCT system to quantify the average strut thickness values of each sample. Strut thickness values were normalized to the designed strut thickness for the corresponding group (Fig. 5D). Results indicate that the average strut diameter was smaller than the nominal designs for each relative density. However, the dimensional variability appeared to be consistent across groups whether the samples were as-printed or post-HIP.

**Fig. 6:**
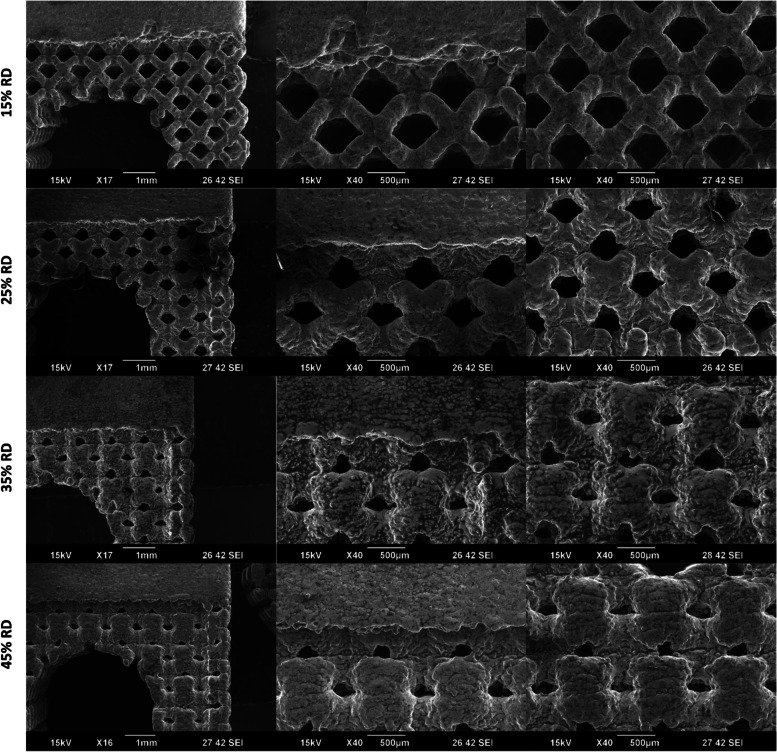
SEM Images of the varying relative densities (RD) printed with the Laser 2 system

As shown in Fig. 6, there are visible structural differences in the lattice coupon with varying relative densities. Despite these visible differences, there is little effect in the measured lattice parameters (Fig. 5) relative to the nominal and compared for differences in variability across all relative density groups.

As stated previously, this analysis began with non-destructive testing to evaluate assessment methods that maintained the structural integrity of each sample. However, due to the conflicting results shown in Figs. 2 and 3, a different and destructive test method (ASTM F1854-15 “Standard Test Method for Stereological Evaluation of Porous Coatings on Medical Implants”) was also used. Cylindrical lattice coupons were evaluated using the microCT method and subsequently the ASTM F1854 method allowing the strut thickness measurements to be compared for the same samples.

**Fig. 7:**
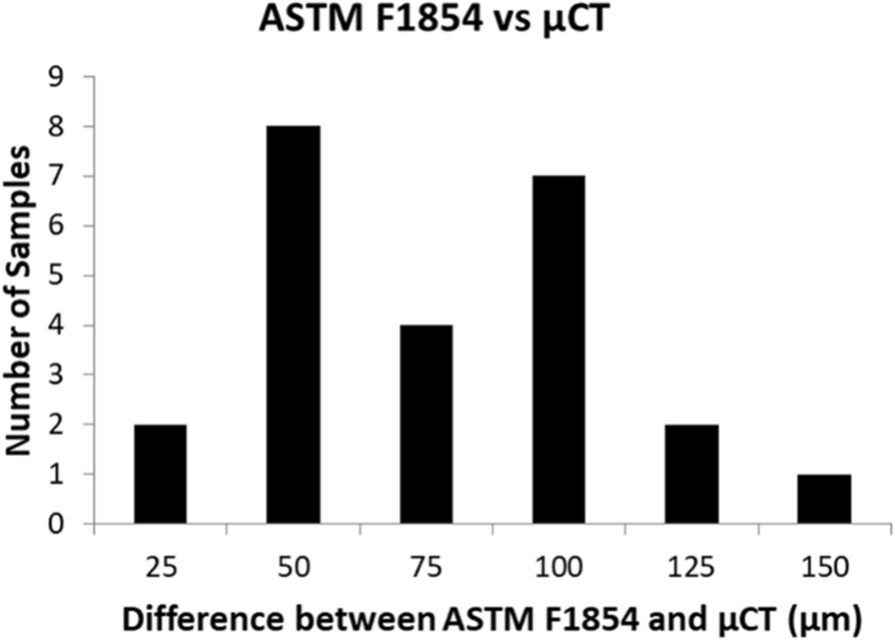
A frequency graph of the additional lattice samples that were used to compare measured strut diameter for the microCT (μCT) method to the ASTM F1854-15 standard. MicroCT results were subtracted from ASTM F1854-15 standard, differences were classified into 25 μm bins

A frequency graph of the difference between the ASTM F1854-15 values and the microCT values are shown in Fig. 7. It was found that the ASTM F1854-15 method gave consistently higher strut diameter values. The average difference between the two methods is 62.9 μm with a maximum difference of 138.4 μm.

## Discussion

This study used test coupons representative of an intervertebral body fusion device, often known as a spinal fusion cage. Aim 1 investigated the effect of design and 3DP technology type by assessing framed and frameless lattice spine cage coupons made using laser and electron beam powder bed fusion and the effect of hot isostatic press post processing. Aim 2 assessed the effect of the lattice relative density for four different densities on the dimensional variability of bulk and lattice dimensions and the effect of hot isostatic press post processing. Aim 3 further investigated the challenges of measuring 3DP lattices and assessed strut thicknesses using microCT and optical measurement techniques.

In Aim 1, results showed differences between printed parts and design files where external dimensions varied up to 700 μm from the nominal. 3DP coupons created using PBF-LB varied by less than 0.2 mm from the nominal designed values. Samples created by EBM varied between 0.4 mm and 0.7 mm from the nominal value. These results are consistent with those found in literature as the Laser 1 measurements were below and the EBM measurements were in line with the maximum average deviation of nearly 0.7 mm from nominal reported for PBF-LB 316 stainless steel [5]. However, the addition of a frame did not show a closer dimensional relationship with the nominal value.

External dimensions were also assessed after a post processing method known as hot isostatic pressing which involves high temperature and pressure to consolidate internal voids and relieve internal stresses. It is unlikely that the consolidation of internal voids will have a significant effect on external dimensions, but relieving internal stresses may affect dimensional stability [21]. It was found that post processing these samples via hot isostatic press appeared to create a statistically significant difference in most part dimensions with the largest difference seen in the Laser 1 length measurements with a difference of 150 μm. The significant change in length for the Laser 1 samples was an interesting finding that was not seen in the EBM samples and may be related to build parameters or differences in material relaxation post-HIP. In some medical devices, such as those that remain static, a difference of 150 μm may not be a clinically relevant deviation, however, in articulating devices with specified tolerancing or devices that need to mate with other components, this difference could be problematic.

When compared to the solid portions, lattice sections of the EBM samples generally deviated farther from nominal than the PBF-LB samples with maximum deviations of more than 0.75 mm and less than 0.1 mm, respectively. These results indicate that, at the current print conditions for the 3DP systems used, the incorporation of lattice structure in general has an increased effect on the external dimensional variability of the EBM lattice coupons compared to the solid portions. As seen in Fig. 3, Laser 1 samples appeared to have different pore areas based on the viewed orientation of the sample. This could potentially be due to differences in pore shape as a result of build parameters. Additionally, the lack of clearly defined struts for the EBM lattice samples is likely a contributing factor to the high dimensional variability for the lattice pore area and strut diameter measurements.

These differences between the Laser 1 and EBM samples are not wholly unexpected given the differences in the 3DP technologies. While the exact performance of a 3DP system is dependent on the make and model, there are published differences between EBM and PBF-LB technologies [22]. In that review article, key process differences were noted: PBF-LB have laser powers ranging from 200 – 1000 W in an inert gas atmosphere operating at 100 – 200 °C while EBM utilizes an electron beam of 3000 W under vacuum operating at 700 – 900 °C. These build parameter differences coupled with different feed stock size leads to EBM generally having a much larger melt pool and surface roughness than PBF-LB while maintaining a faster build rate [22]. With the difference in melt pool size, beam power, and operating temperatures the difference in dimensional variability, both in magnitude and in lattice/solid difference, is not surprising.

Analyzing the combined results from Figs. 2 and 3 (external dimensions, pore area, etc.), it was unexpected that the strut diameter would be smaller than the designed value given that the average pore area is below the nominal and the external dimension are mainly above the nominal. With a smaller pore area, it would be expected that the strut diameters would be larger than the nominal to compensate. These results indicate a possible need for further investigation into the assessment methods. Currently, non-destructive assessment techniques have been used for all measurements, but combining measurement results indicates that one of the sets of measurements may be inconsistent. Given the macro dimensions were obtained using calibrated calipers, it is reasonable that either the pore area or strut diameter measurements may be the source of this inconsistency. This led to a more detailed investigation of existing strut diameter measurement methods which is discussed in Aim 3.

Direct comparisons between laser and EBM samples were not conducted due to the limitations that result from commercial sourcing of samples. Potential advances in technology, software and controls, and printing mechanisms could provide sources of variation between PBF-LB and EBM printing methodologies. Additionally, specific print parameters, such as build orientation, were not provided from all commercial sources rendering direct comparisons impractical.

In Aim 2, results indicated that relative density generally had little impact on the external dimensional variability for the width and depth dimensions of as-printed samples as shown in Fig. 5A(i) with only one statistically significant difference observed between two of the relative density groups for the length dimension. This generally implies that once the dimensional variability of a lattice structure has been established it is unlikely that altering the relative density will change the overall external dimensions.

Statistically significant differences were found after the HIP process compared to the as-printed state. Interestingly, part lengths were decreased by 150–200 μm for all relative density groups which is similar to the post-HIP length changes seen in Aim 1. These results are interesting because a similar decrease was seen across two different manufacturers which could be due to similar build parameters or stress relaxation after the HIP process. As previously mentioned, this dimensional change could be significant in some medical device applications.

When compared to the lattice portions of the samples, the solid base sections of the Laser 2 samples appear to be less prone to dimensional variability. This was observed for all relative densities with a slight convergence in lattice dimensions as the relative density increases. As seen in Fig. 6, with increasing relative density, the edge of the lattice became flatter. This is likely responsible for the change in lattice behavior seen in Fig. 5B.

A confounding measurement in the pore sizes was observed in Fig. 5C as the pore values were consistently above the nominal for Laser 2 samples. This is not consistent with the trends observed in Fig. 3A where pore sizes generally trended below the nominal for Laser 1 and EBM samples. While this could be due to differences in the 3DP technology, the authors hypothesize that this may be due to difference in build parameter optimizations for this given feature size. This underscores the need to investigate the effect of build parameters on lattice dimensions.

Throughout this study, discrepancies in strut thickness values were found based on measurement method. Aim 3 sought to assess the difference in measured strut diameter between two commonly used evaluation methods. The difference in strut diameter measurements of the same samples varied between microCT and ASTM F1854-15 by as much as 138.4 μm, a 40.6% difference. This raises serious questions of strut thickness fidelity when evaluating struts in the 200–300 μm range as this deviation could be up to 50% of the nominal strut thickness. This large deviation indicates that the commonly used and widely accepted assessment methods may not correspond as they do not produce the same or even similar measured values. A consequence of this finding is that the dimensional inconsistencies seen in Figs. 2 and 3 could be explained by divergence microCT measurements of the strut thickness from established stereological methods. This indicates a need for standardization of more robust evaluation and quantification tools to ensure accurate dimensional results for 3DP lattices.

## Conclusion

Based on the results and discussion of the two parts of this work, there were a number of conclusions. 3DP coupons created by both PBF-LB and EBM differed from the nominal design for both bulk and lattice measurements. The addition of a frame did not show a closer dimensional relationship with the nominal value for either the PBF-LB and EBM samples. Post processing these samples via hot isostatic press appeared to impact the length dimensions more than any other dimension for PBF-LB samples which was observed in both parts of this study.

Additionally, lattice portions of the EBM samples showed larger dimensional deviations than the solid base when compared to nominal. Varying relative density had little impact on the external dimensional variability for the as-printed state for width and depth dimension (*p* > 0.05) but did have a statistically significant impact on the length dimension (*p* < 0.05). A divergence in strut thicknesses, up to 40.6%, was found between microCT and ASTM F1854-15 which indicates the need for a more robust lattice measurement protocol.

Overall, it should be noted that the 3D Printed parts can deviate from the nominal design and post processing may produce varying dimensional outcomes. Part tolerancing and device application should be considered when designing specific lattice-containing medical devices.

## Data Availability

The datasets used and/or analyzed during the current study are available from the corresponding author on reasonable request.

## Abbreviations

3DP: 3-Dimensional Printing
AM: Additive Manufacturing
DT: Direct Transformation
EBM: Electron Beam Melting
HIP: Hot isostatic press
PBF-LB: Laser-based powder bed fusion
SEM: Scanning electron microscopy
μCT/microCT: Micro computed tomography

## References

[1] Di Prima M, Coburn J, Hwang D, Kelly J, Khairuzzaman A, Ricles L (2016). Additively manufactured medical products–the FDA perspective.. 3D print me.

[2] Technical Considerations for Additive Manufactured Medical Devices. In: Research UFaDACfDaRHaCfBEa, editor. https://www.fda.gov/regulatory-information/search-fda-guidance-documents/technical-considerations-additive-manufactured-medical-devices2017.

[3] Ricles LM, Coburn JC, Di Prima M, Oh SS (2018). Regulating 3D-printed medical products. Sci transl med.

[4] Makes AAMSC (2018). Standardization Roadmap for Additive Manufacturing-Version 2.0. Am Makes ANSI Addit Manuf Stand Collab.

[5] Lieneke T, Adam G, Leuders S, Knoop F, Josupeit S, Delfs P (2015). Systematical determination of tolerances for additive manufacturing by measuring linear dimensions.

[6] Lieneke T, Denzer V, Adam GA, Zimmer D (2016). Dimensional tolerances for additive manufacturing: Experimental investigation for Fused Deposition Modeling. Procedia CIRP.

[7] Rupal BS, Anwer N, Secanell M, Qureshi AJ (2020). Geometric tolerance and manufacturing assemblability estimation of metal additive manufacturing (AM) processes. Mater Des.

[8] Dantan J-Y, Huang Z, Goka E, Homri L, Etienne A, Bonnet N (2017). Geometrical variations management for additive manufactured product. CIRP Ann.

[9] Zuowei Z, Keimasi S, Anwer N, Mathieu L, Lihong Q (2017). Review of shape deviation modeling for additive manufacturing.

[10] Cheng L, Tsung F, Wang A (2017). A statistical transfer learning perspective for modeling shape deviations in additive manufacturing. IEEE Robotics and Automation Letters.

[11] Brajlih T, Valentan B, Balic J and Drstvensek I. Speed and accuracy evaluation of additive manufacturing machines. Rapid Prototyping J. 2011;17(1):64–75.

[12] Minetola P, Calignano F, Galati M (2020). Comparing geometric tolerance capabilities of additive manufacturing systems for polymers. Addit Manuf.

[13] Fotovvati B, Asadi E (2019). Size effects on geometrical accuracy for additive manufacturing of Ti-6Al-4V ELI parts. The International Journal of Advanced Manufacturing Technology.

[14] Moroni G, Syam WP, Petro S (2014). Towards early estimation of part accuracy in additive manufacturing. Procedia CIRP.

[15] Toth T, Hudak R, Zivcak J (2015). Dimensional verification and quality control of implants produced by additive manufacturing. Quality Innovation Prosperity.

[16] Jin SJ, Kim DY, Kim JH, Kim WC (2019). Accuracy of Dental Replica Models Using Photopolymer Materials in Additive Manufacturing: In Vitro Three-Dimensional Evaluation. J Prosthodont..

[17] Salmi M, Paloheimo K-S, Tuomi J, Wolff J, Mäkitie A (2013). Accuracy of medical models made by additive manufacturing (rapid manufacturing). Journal of Cranio-Maxillofacial Surgery.

[18] Navarro J, Din M, Janes ME, Swayambunathan J, Fisher JP and Dreher ML. Effect of print orientation on microstructural features and mechanical properties of 3D porous structures printed with continuous digital light processing. Rapid Prototyping J. 2019;25(6):1017–29.

[19] Wang MO, Piard CM, Melchiorri A, Dreher ML, Fisher JP (2015). Evaluating changes in structure and cytotoxicity during in vitro degradation of three-dimensional printed scaffolds. Tissue Eng Part A.

[20] Shipley H, McDonnell D, Culleton M, Coull R, Lupoi R, O’Donnell G, et al. Optimisation of process parameters to address fundamental challenges during selective laser melting of Ti-6Al-4V: A review. Int J Mach Tools Manuf. 2018;128:1–20.

[21] Du Plessis A, Yelamanchi B, Fischer C, Miller J, Beamer C, Rogers K (2021). Productivity enhancement of laser powder bed fusion using compensated shelled geometries and hot isostatic pressing. Advances in Industrial and Manufacturing Engineering.

[22] Bhavar V, Kattire P, Patil V, Khot S, Gujar K, Singh R. A review on powder bed fusion technology of metal additive manufacturing. Additive manufacturing handbook; 2017. p. 251–253.

